# Supplementary material for: Application of an O-Linked Glycosylation System in Yersinia enterocolitica Serotype O:9 to Generate a New Candidate Vaccine against Brucella abortus
Source: Microorganisms. 2020 Mar 20;8(3):436. doi: 10.3390/microorganisms8030436 (PMC7143757; doi:10.3390/microorganisms8030436)
Supplement: Supplementary file 1 [file microorganisms-08-00436-s001.zip › Supplementary Figures Microorganisms/Figure S1.pdf]

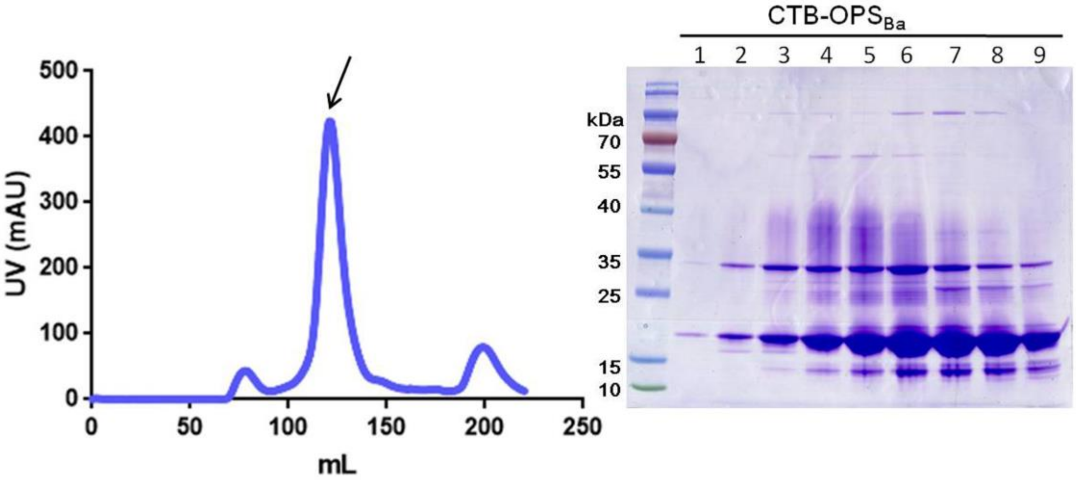

Figure S1. Purification of CTB-OPS$_{Ba.}$ Left: Size exclusion chromatography of CTB-OPS$_{Ba}$. The black arrow represents the target glycoprotein peak. Right: Nine collected fractions were analysed by Coomassie Blue staining, then fractions 3-6 (from left to right) were concentrated.
