# Supplementary material for: Application of an O-Linked Glycosylation System in Yersinia enterocolitica Serotype O:9 to Generate a New Candidate Vaccine against Brucella abortus
Source: Microorganisms. 2020 Mar 20;8(3):436. doi: 10.3390/microorganisms8030436 (PMC7143757; doi:10.3390/microorganisms8030436)
Supplement: Supplementary file 1 [file microorganisms-08-00436-s001.zip › Supplementary Figures Microorganisms/Figure S2.pdf]

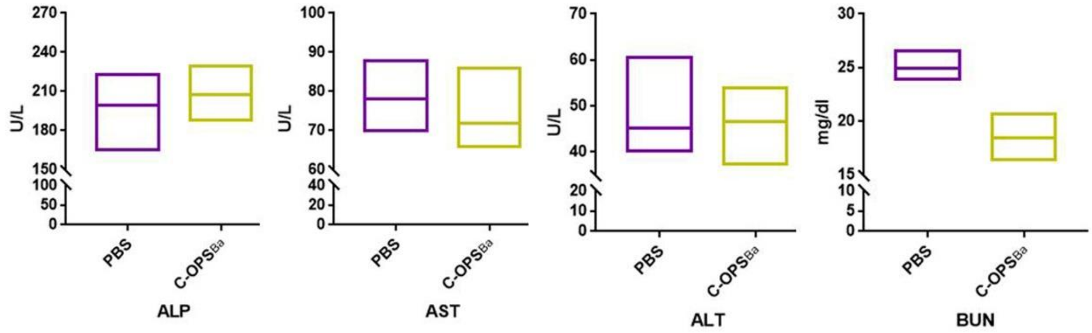

Figure S2. Biochemical indicators in the sera of mice after three immunizations. Serum biochemical indicators were measured in C-OPS$_{Ba}$- and PBS-immunized mice after the third immunization. ALP: alkaline phosphatase; AST: aspartic transaminase; ALT: alanine aminotransferase; BUN: blood urea nitrogen. The results were all within normal range. The markers analysed are representative indicators of liver and kidney function, and abnormal values indicate that the hepatobiliary system and the renal systems are damaged. Results are expressed as the means of measured values (n=5).
