# Supplementary material for: Application of an O-Linked Glycosylation System in Yersinia enterocolitica Serotype O:9 to Generate a New Candidate Vaccine against Brucella abortus
Source: Microorganisms. 2020 Mar 20;8(3):436. doi: 10.3390/microorganisms8030436 (PMC7143757; doi:10.3390/microorganisms8030436)
Supplement: Supplementary file 1 [file microorganisms-08-00436-s001.zip › Supplementary Figures Microorganisms/Figure S3.pdf]

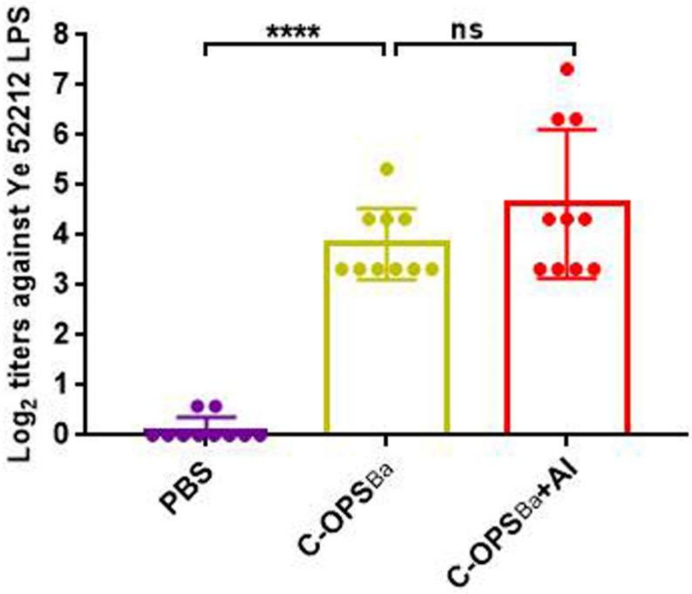

Figure S3. IgG responses against YeO9_52212 LPS. IgG titres against 52212 LPS were measured in the sera of PBS-, C-OPS$_{Ba}$- and C-OPS$_{Ba}$+Al-vaccinated mice after three immunizations. Each value represents the mean ± standard deviation of log_2-transformed titres in the sera of individual mice (shown as points) from each group. The unpaired t-test was used to evaluate differences between IgG titres (****, *P*<0.0001; ns, no statistically significant difference).
