# Supplementary material for: Application of an O-Linked Glycosylation System in Yersinia enterocolitica Serotype O:9 to Generate a New Candidate Vaccine against Brucella abortus
Source: Microorganisms. 2020 Mar 20;8(3):436. doi: 10.3390/microorganisms8030436 (PMC7143757; doi:10.3390/microorganisms8030436)
Supplement: Supplementary file 1 [file microorganisms-08-00436-s001.zip › Supplementary Figures Microorganisms/Figure S4.pdf]

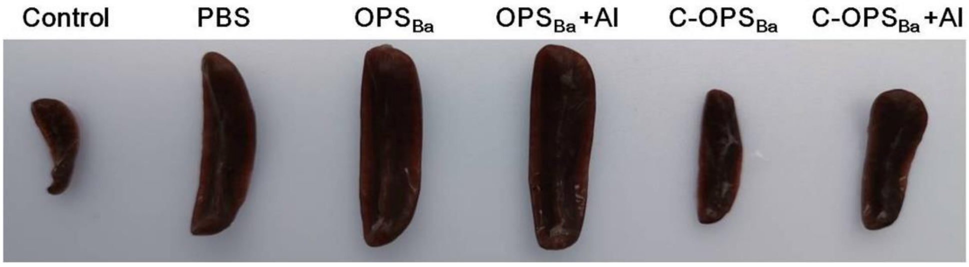

Figure S4. Spleen sizes of control (normal) and immunized mice following infection with *B. abortus* A19. On the 10th day following non-lethal A19 challenge, spleens of mice in each group were removed and compared with those of normal mice to observe differences in size and shape.
