# Supplementary material for: Application of an O-Linked Glycosylation System in Yersinia enterocolitica Serotype O:9 to Generate a New Candidate Vaccine against Brucella abortus
Source: Microorganisms. 2020 Mar 20;8(3):436. doi: 10.3390/microorganisms8030436 (PMC7143757; doi:10.3390/microorganisms8030436)
Supplement: Supplementary file 1 [file microorganisms-08-00436-s001.zip › Supplementary Figures Microorganisms/Figure S5.pdf]

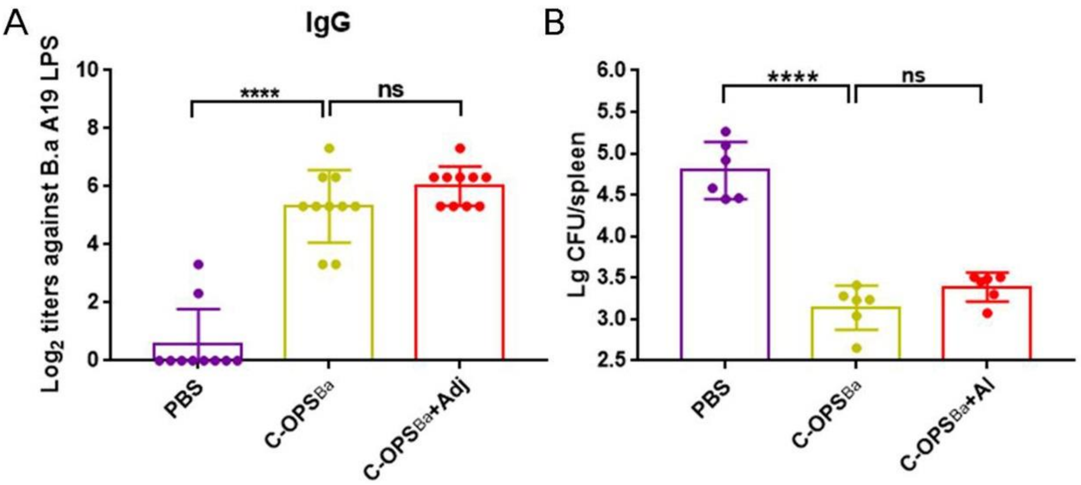

Figure S5. IgG responses against *B. abortus* A19 LPS and bacterial loads in the spleens of PBS-, C-OPS$_{Ba}$- and C-OPS$_{Ba}$+Al-vaccinated mice following infection with a higher dose of *B. abortus* A19. Mice were immunized with vaccines or PBS as described above. (A) IgG titres against A19 LPS in the sera of mice were measured on 10th day following three immunization s(n=10). Each value represents the mean ± standard deviation of log$_2$-transformed titres in the sera of individual mice (shown as points) from each group. (B) Mouse spleens were collected 7 days following infection with 4.16×10^7 CFU A19 (about three times the dose mentioned above in legend of Figure 3.; the survival rate of PBS-vaccinated mice was 80% after being challenged with this dose of A19). The total bacterial loads were calculated (n=6). Each value represents the mean ± standard deviation of log$_{10}$-transformed bacterial loads (CFU/spleen) of individual mice (shown as points) from each group. The unpaired t-test was used to evaluate differences between IgG titres and bacterial loads (****, *P*<0.0001; ns, no statistically significant difference).
